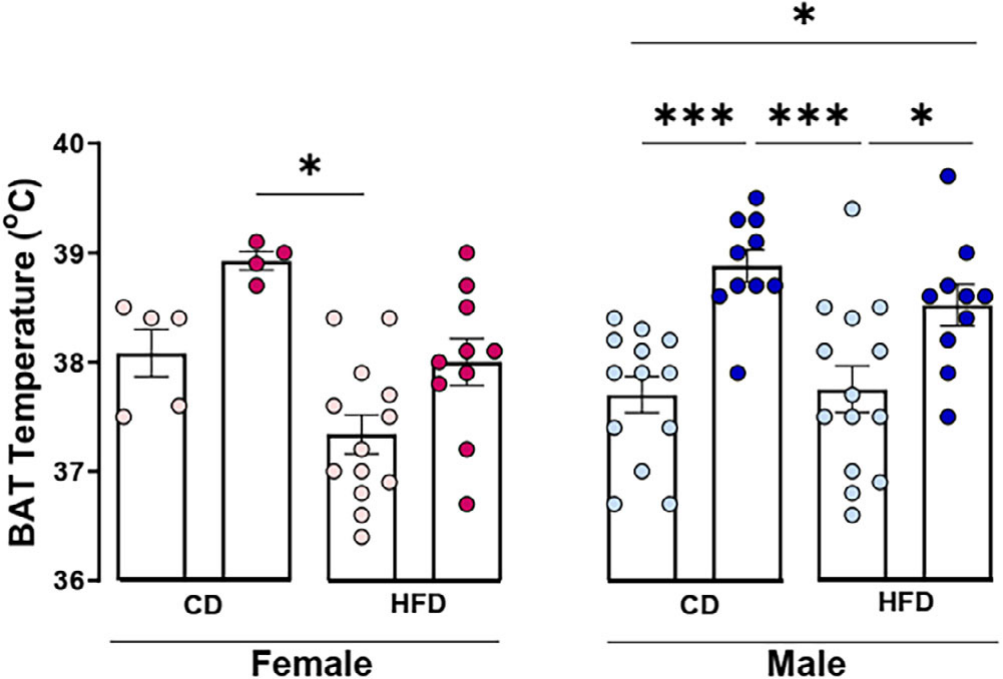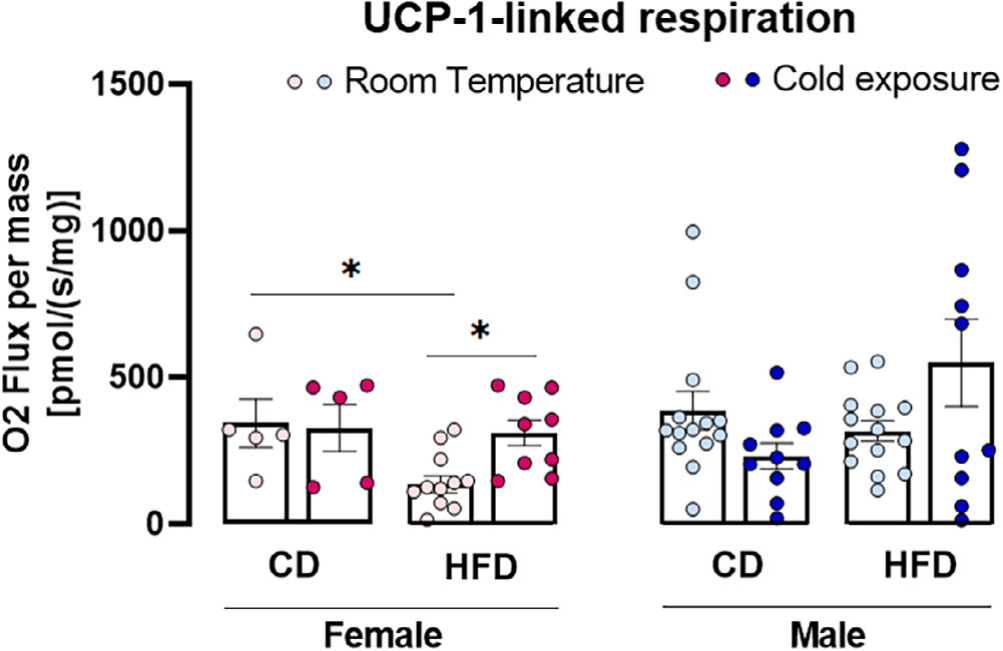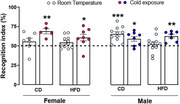# Intermittent cold exposure reversed high‐fat diet‐induced metabolic and cognitive dysfunctions in mice

**DOI:** 10.1002/alz.089267

**Published:** 2025-01-03

**Authors:** Wellinghton de Medeiros Barros, Paula Maria Bellozi, Maria Luiza Soccio Bezerra, Daniel Domingues, Louise Tavares Garcia Pereira, Jair Trapé Goulart, Angélica Amorim Amato, Andreza Fabro de Bem, Laboratory of Bioenergetics and Metabolism – Department of Physiological Sciences, University of Brasília – Brasília/DF, Brazil

**Affiliations:** ^1^ Universidade de Brasília, Brasília, Federal District Brazil; ^2^ Universidade de Brasília, Brasília Brazil

## Abstract

**Background:**

Recent research has demonstrated that the consumption of high fat diet (HFD) can lead to metabolic dysfunctions and cognitive impairments in both mice models and humans. Given the potential negative effects of HFD, it is crucial to explore non‐pharmacological alternatives that can serve as a potential treatment for both metabolic dysfunctions and behavioral effects induced by HFD. Therefore, the aim of this study is to assess the impact of chronic and intermittent exposure to cold temperature on the metabolic and cognitive changes associated with HFD consumption.

**Methods:**

Male and female C57Bl/6 adult mice were divided into 8 groups exposed to different diets and temperature conditions (HFD or control diet (CD) and, cold (4oC) or room temperature (RT) (22oC) exposure for 10 weeks). The ∆ of body mass, brown adipose tissue (BAT) temperature and oxygen consumption as well as behavior were evaluated after the experimental period.

**Results:**

HFD increased the body mass in females (CD+RT x HFD+Cold p = 0.02) and males (CD+RT x HFD+RT p < 0.0001), however, cold exposure was able to reverse this metabolic outcome (male HFD+RT x HFD+Cold p = 0.01). Cold exposure increased BAT temperature (female: CD+Cold x HFD+RT p = 0.0005; male CD+RT x CD+Cold p = 0.0005, CD+RT x HFD+Cold p = 0.024, HFD+RT x HFD+Cold p = 0.035). Regarding oxygen consumption, UCP‐1‐linked respiration decreased in the female mice fed a HFD (CD+RT x HFD+RT p = 0.04), however, cold exposure reverted this effect (HFD+RT x HFD+Cold p = 0.03). Interestingly, we observed that the consumption of HFD caused recognition memory deficits in both female (p = 0.0728) and male (p = 0.51) mice that were reversed by cold exposure (female: p = 0.04, and male: p = 0.002).

**Conclusions:**

With the present data we can infer that cold exposure can increase the activity of BAT, thereby mitigating the harmful effects of HFD on cognition and metabolism. In summary, chronic and intermittent cold exposure has proven capable of attenuating the deleterious metabolic and cognitive effects induced by HFD consumption in mice.